# Accelerated junctional rhythm (AJR) revealing light-chain cardiac amyloidosis: A case report with literature review

**DOI:** 10.1016/j.amsu.2022.103410

**Published:** 2022-03-03

**Authors:** Raid Faraj, Zaineb Bourouhou, Sidaty Oussama, Asmaa Bouamoud, Hasna Rami, Amina Samih, Ibtissam Fellat, Jamila Zarzur, Mohamed Cherti

**Affiliations:** Mohammed V University, Rabat, Morocco

**Keywords:** Cardiac amyloidosis, Light-chain, Junctional rhythm, Cardiac arrythmias, Heart failure

## Abstract

**Introduction and importance:**

Cardiac amyloidosis (CA) is a rare condition, characterized by fibrillary proteins infiltration in the extracellular space of the heart. Even though many types of cardiac amyloidosis exist, light chain amyloidosis (AL) and transthyretin amyloidosis (ATTR) remain the most described forms. The diagnosis of amyloidosis represents a real challenge for clinicians, requiring both invasive and non-invasive investigations. Conduction defects and atrial arrhythmias are well known complications of cardiac amyloidosis. However, only a few studies have reported junctional rhythm a primary presentation of light chain cardiac amyloidosis (AL). An early diagnosis and proper management are crucial to improve the prognosis of this disease.

**Case presentation:**

Here, we report a rare case of a 48 year-old patient, in acutely decompensated heart failure, presenting an accelerated junctional rhythm (AJR) as initial presentation of light-chain cardiac amyloidosis. The diagnosis was made based on clinical, biological, radiological and histological findings. This case shows diagnostic difficulties and management of this rare disease.

## Highlights

• Cardiac amyloidosis is a rare condition, characterized by fibrillary proteins infiltration in the extracellular space of the heart.
• Junctional rhythm a primary presentation of light chain cardiac amyloidosis (AL) is extremely rare.
• The association between cardiac AL amyloidosis and heart failure without etiologic treatment can be dramatic with an overall median survival of six months.

## 1 Introduction

Cardiac amyloidosis (AL) is an infiltrative cardiomyopathy, resulting from the deposition of immunoglobulin light chains. Its incidence is in constant increase during the last years among individuals aged between 18 and 64 years [1]. Many patients present a conduction disorder during the evolution of this disease but accelerated junctional rhythm (AJR) as an initial presentation of cardiac amyloidosis is less recognized and described. Recognizing cardiac amyloidosis (AL) at an early stage is essential, as it allows the initiation of early treatment and oftentimes improves of the outcome. Indeed, the association between cardiac AL amyloidosis and heart failure without etiologic treatment can be dramatic with an overall median survival of six months [2].

To the best of our knowledge, it's the first case of accelerated junctional rhythm (AJR) revealing light chain cardiac amyloidosis among a young patient with no medical history.

Our case report was written according to CARE guidelines [3].

## 2 Case presentation

A 48 year old patient presented with worsening dyspnea on exertion with orthopnea associated to lower-extremity edema that appeared 2 weeks before his admission.

**Fig. 1:**
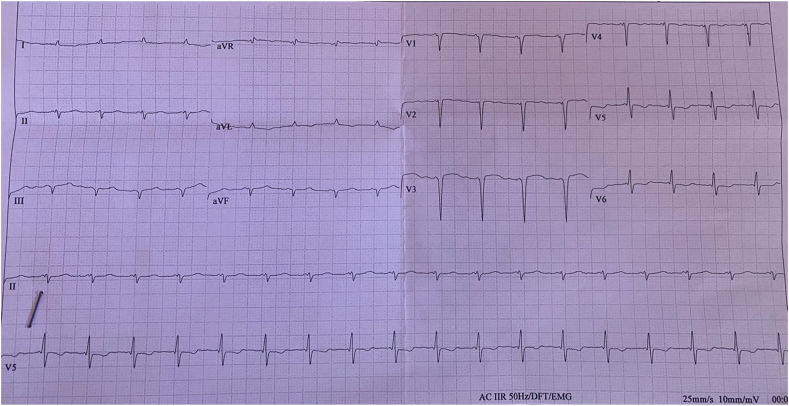
The initial ECG showing an accelerated junctional rhythm at 96 bpm with a pseudo-infarct pattern in V1–V3.

**Fig. 2:**
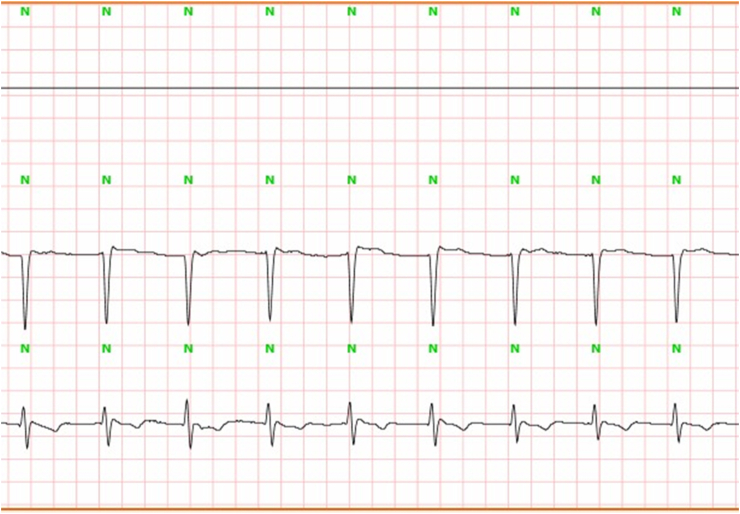
Holter ECG showing an accelerated junctional rhythm (AJR).

**Fig. 3:**
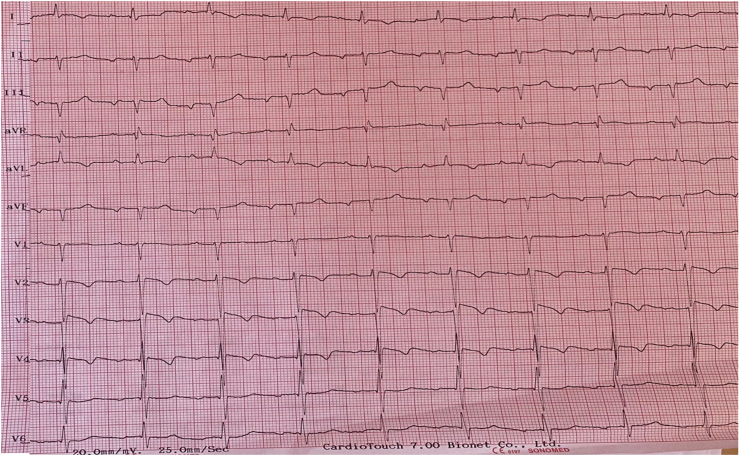
High voltage ECG of the admission showinga coronary sinus rhythm with a first-degree atrioventricular block (PR interval >200 ms), low voltage in limb leads and a pseudo-infarct pattern.

**Fig. 4:**
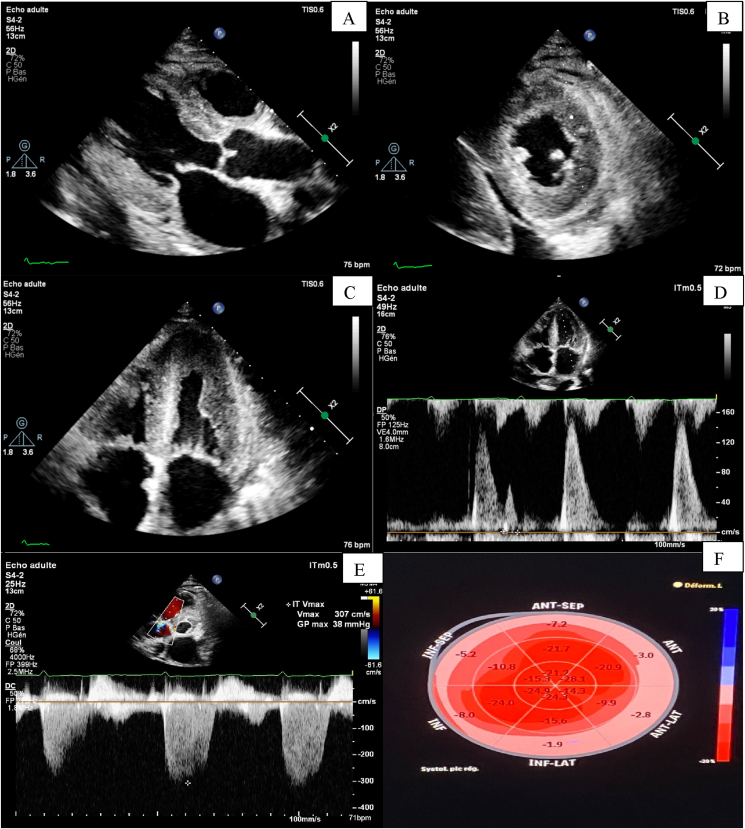
TEE findings: (A) parasternal long axis view showing shiny aspect of the interventricular septum with concentric wall thickening and a mild pericardial effusion. (B) parasternal short axis view showing an important concentric wall thickening of the left ventricle (C) Apical long axis view showing a biatrial enlargement associated with biventricular hypertrophy (D) Pulsed wave doppler of mitral inflow suggesting a restrictive mitral inflow pattern. (E) 2D-Strain showing the “bulls eye’’ appearance (F).

**Fig. 5:**
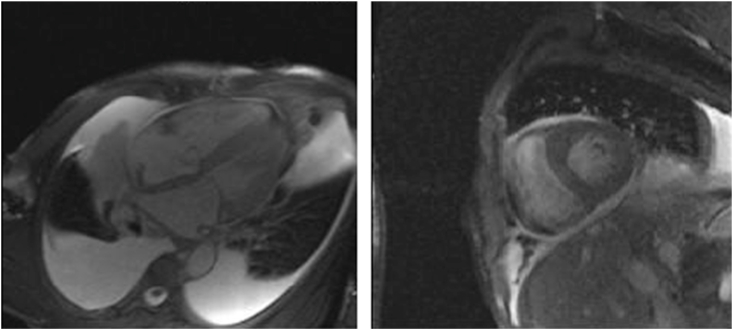
CMR showing delayed post gadolinium enhancement of myocardium, suggesting amyloid deposition in the myocardium.

**Fig. 6:**
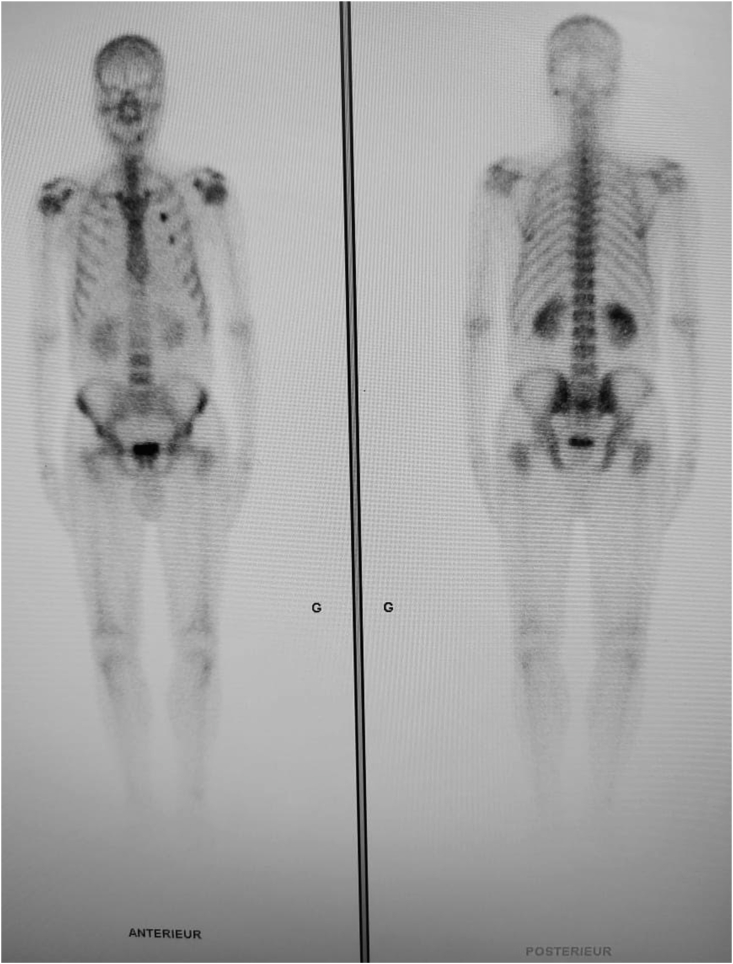
Bone scintigraphy showing the absence of cardiac uptake and suggestive of light-chain cardiac amyloidosis.

He did not report any similar family medical history. The general examination found a conscious patient. His heart rate was 70 b/m, his blood pressure was 136/75 mm Hg. He was polypneic and orthopneic with a respiratory rate of 28 breaths/min, an O2 saturation of 96% on ambient air. Also noted, fine bibasilar crackles with 4+ pitting edema bilaterally. Further physical examination was normal. A 12-lead electrocardiogram (Fig. 1) and Holter ECG (Fig. 2), performed 1 week before his admission, revealed the presence of an accelerated junctional rhythm at 98 bpm with a short PR interval (<120 ms) indicating a junctional rather than atrial focus and a pseudo-infarct pattern. Another one was performed in our department revealing this time a coronary sinus rhythm with a first-degree atrioventricular block (PR interval >200 ms), low voltage in limb leads and a pseudo-infarct pattern (Fig. 3). Initial laboratory data noticed high levels of NT-pro BNP and cardiac Troponin (respectively 3525 pg/ml and 89 ng/l). The rest of laboratory exams was normal. A transthoracic echocardiogram revealed grainy and shiny aspect of the interventricular septum with concentric wall thickening and biatrial enlargement with mild pericardial effusion (Fig. 4-A). Ejection fraction was at 55%. Impaired relaxation and elevated filling pressures with restrictive mitral inflow pattern were noted, indicating severe diastolic dysfunction (Fig. 4-C). Pulmonary artery systolic pressure (PASP) was estimated to be 58 mmHg assuming a right atrial pressure of 20 mmHg (Fig. 4-E). In addition to that, the echocardiographic strain imaging revealed the “bullseye’’ appearance (Fig. 4-F), consistent with preserved longitudinal systolic function at the LV apex in contrast with reduced longitudinal shortening at basal level. Coronary angiography was performed and revealed no coronary artery stenosis. Since clinical, echocardiographic and biological findings were strongly suggestive for amyloidosis, cardiac magnetic resonance imaging (MRI) was performed. It objectivated delayed postgadolinium enhancement of myocardium, suggesting amyloid deposition in the myocardium (Fig. 5). The diagnosis of cardiac amyloidosis was highly considered, the patient underwent a salivary gland biopsy, which confirmed the presence of amyloid deposits. Further investigations were realized to determine the type of cardiac amyloidosis, including serum protein immunofixation, serum Kappa/Lamda free light chain ratio analysis and urine protein immunofixation. A gamma-peak was observed and Kappa/Lambda ratio reduced to 0.15. Bone scintigraphy did not show signs in favor of TTR amyloidosis (Fig. 6), thus confirming the diagnosis of AL amyloidosis.

The differential diagnosis includes the other etiologies of heart failure with preserved ejection fraction (HFpEF), left ventricle hypertrophy which is associated with cardiac hypertrophic cardiomyopathy, hypertension or Fabry disease.

**Fig. 7:**
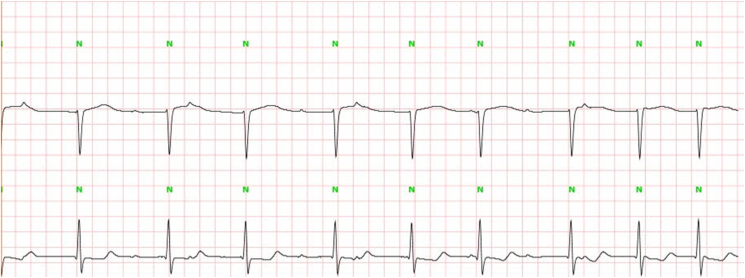
Holter ECG showing a complete atrioventricular block.

The initial outcome was favorable with regression of the signs of heart failure under intravenous diuretic treatment. During his first week's hospitalization, the patient presented a syncope related to a complete atrioventricular block (Fig. 7), reason why he benefited from an implantation of a double chamber pacemaker. The patient, after improvement of his cardiac decompensation, was transferred to hematology department to initiate urgent chemotherapy directed against plasma-cells.

## 3 Discussion

Amyloidosis is considered as a rare infiltrative disease, characterized by deposition of fibrillary proteins in the extracellular space that concerns different organs such as the kidney, heart, liver and the autonomic nervous system [4].Cardiac involvement in amyloidosis can be objectified in different types of amyloidosis but it is important to emphasize that 95% of the cases are caused by light-chain amyloidosis (AL) and transthyretin amyloidosis (ATTR) [5,6].

As mentioned in the 2021 ESC myocardial working group position paper, cardiac amyloidosis must be suspected when left ventricle wall thickness ≥12mm is associated with ≥1 Red flag or clinical scenario that include cardiac or extracardiac signs and symptoms [7]. Diagnosing cardiac amyloidosis remains a real challenge for clinicians but the emergence of new imaging techniques (such as cardiac MRI and bone scintigraphy) and of tissue biopsy has made the diagnosis lately more obvious.

This infiltrative disease can lead to different complications, at the top of the list: heart failure in advanced stages, conduction defects and atrial arrhythmias [8,9]. However, few data is available to implicate the role of cardiac amyloidosis as the cause of junctional rhythm. In a recent article published in the International Journal of Cardiovascular Sciences, authors have reported junctional rhythm as a possible complication of light-chain cardiac amyloidosis and multiple myeloma in a 80 year old female [8]. Unexplained left ventricular hypertrophy associated with heart failure was present in both our case reports, and should therefore indicate possible cardiac involvement of amyloidosis.

Cardiac arrhythmias are very common during the evolution of cardiac amyloidosis and represent an important cause of morbidity and mortality [10]. In a recent study including 359 patients, Boldrini et al. noted a high prevalence of conduction disease among patients with cardiac AL amyloidosis which is consistent with the case of our patient, who developed syncope during his hospitalization due to a complete atrioventricular block.

Different staging systems have been proposed for cardiac amyloidosis. The most used for AL amyloidosis is the revised Mayo stage system which is based on levels of NT-proBNP, cardiac Troponin and free light chains [11]. In our case, the patient was considered in stage III of his disease (2 parameters) with a 5-year survival estimated at 28%.

Treatment of complications and of the underlying cause is the basis of cardiac amyloidosis management. While loop diuretics are considered as the backbone therapy of heart failure in cardiac amyloidosis along with control fluid, the use of beta blockers and angiotensin-converting enzyme inhibitors is not beneficial and may be even harmful, despite their efficacy in the treatment of other types of systolic heart failure [12].

Even though, sudden cardiac death is frequent in patients suffering from cardiac AL amyloidosis, there is no scientific evidence supporting the use of implantable cardioverter defibrillator (ICD) for primary prevention [13]. For patients who develop conduction system disease, general indications for cardiac pacing should be applied.

Furthermore, the specific therapy for AL amyloidosis involves the administration of chemotherapy and/or autologous stem cell transplantation. The most satisfactory results are noted with the use of the combination of Bortezomib, Dexamethasone and Cyclophosphamide [12].

## 4 Conclusion

Cardiac involvement could be the sole and primary presentation of amyloidosis.

Clinicians must be aware of this and should consider this disease in patients with unexplained wall thickness in the presence of cardiac and/or extra-cardiac red flags and/or in particular clinical situations. Early diagnosis and proper management are essential to improve the prognosis of this deadly disease.

## Learning objectives

1) To be able to make positive and etiological diagnosis of cardiac amyloidosis.
2) To understand cardiac arrhythmias secondary to cardiac amyloidosis.

## Ethical approval

The ethical committee approval was not required give the article type (case report).However, the written consent to publish the clinical data of the patients was given and is available to check by the handling editor if needed.

## Sources of funding for your research

None.

## Author contribution

Raid Faraj: Study concept, Data collection, Data analysis, Writing the paper.

Bourouhou Zaineb: Data collection, Data analysis.

Sidaty Oussama: Data collection.

Asmaa Bouamoud: Data collection.

Hasna Rami: Data collection.

Amina Samih: Data collection.

Ibtissam Fellat: Supervision and data validation.

Jamila Zarzur: Supervision and data validation.

Cherti Mohamed: Supervision and data validation.

## Registration of research studies

This is not an original research project involving human participants in an interventional or an observational study but a case report. This registration is was not required.

## Guarantor

Raïd Faraj.

## Consent

Obtained.

## Provenance and peer review

Not commissioned, externally peer-reviewed.

## Declaration of competing interest

None.
